# Bio-Characterization and Liquid Chromatography–Mass Spectrometry Analysis of Exopolysaccharides in Biofilm-Producing Cyanobacteria Isolated from Soil Crust: Exploring the Potential of Microalgal Biomolecules

**DOI:** 10.3390/biology12081065

**Published:** 2023-07-30

**Authors:** Mani Vinoth, Sivaprakasam Sivasankari, Abdul Kareem Khaleel Ahamed, Khawla Ibrahim Alsamhary, Nouf Mohammed Al-enazi, Neveen Abdel-Raouf, Reem Mohammed Alharbi, Rasiravathanahalli Kaveriyappan Govindarajan, Gangalla Ravi, Khaloud Mohammed Alarjani, Essam N. Sholkamy

**Affiliations:** 1 PG and Research Department of Botany, Jamal Mohamed College, Tiruchirappalli 620020, Tamil Nadu, Indiacdrakjmc@gmail.com (A.K.K.A.); 2 Department of Biology, Periyar Government Arts College, Cuddalore 607001, Tamil Nadu, India; san.s41190@gmail.com; 3 Departmen of Biology, College of Science and Humanities in Al-Kharj, Prince Sattam Bin Abdulaziz University, Al-Kharj 11942, Saudi Arabia; k.alsamhary@psau.edu.sa (K.I.A.); n.alenazi@psau.edu.sa (N.M.A.-e.); n.khaliefa@psau.edu.sa (N.A.-R.); 4 Botany and Microbiology Department, Faculty of Science, Beni-Suef University, Salah Salem Street, Beni-Suef 62511, Egypt; 5 Biology Department, Science College, University of Hafr Al Batin, Hafr Al Batin 39524, Saudi Arabia; rmalharbi@uhb.edu.sa; 6 Department of Biotechnology, Yeungnam University, Gyeongsan 38541, Gyeongbuk, Republic of Korea; biogovindarajan@gmail.com; 7 Department of Microbiology, Kakatiya University, Warangal 506009, Telangana, India; g.ravi320@gmail.com; 8 Department of Botany and Microbiology, College of Science, King Saud University, Riyadh 11451, Saudi Arabia

**Keywords:** biological soil crust, cyanobacteria, antioxidant, exopolysaccharide, flocculation

## Abstract

**Simple Summary:**

In sacred forest groves, biological soil crusts comprise cyanobacteria covering the surface of the soil. Extracellular polysaccharides (EPSs) from the biological soil crust enhance the availability of nutrients and water. This study aimed to extract and characterize EPSs from the biological soil crust, in addition to gaining insights into their potential as a bioflocculant and antioxidant. The cyanobacteria diversity in the biological crust and their phylogenetic relationships in response to the forest ecosystem are addressed. The biochemical analysis of EPSs revealed an abundance of carbohydrates and proteins. FTIR and LC-MS profiles of EPSs revealed various functional groups which confer antioxidant and flocculation activity. EPS-releasing biological soil crust acts as a biofertilizer in sacred forests by contributing carbon, nitrogen, soil aggregation, and moisture retention.

**Abstract:**

Exopolysaccharide-producing cyanobacterial strains in biological soil crusts are described, in addition to their chemical properties and antioxidant and flocculation activities. The EPSs from Pudukkottai blackish biological soil crusts (PBBSCs) showed significant amounts of total soluble proteins (0.1687 mg/mL) and carbohydrates (0.8056 mg/mL) compared with the Ariyalur blackish biological soil crusts (ABBSCs). LC-MS analysis of the cyanobacterial polysaccharides revealed the presence of natural sugars such as ribose and glucose/mannose, and uronic acids. The FTIR spectrum showed specific peak for OH and –NH stretching, C–H stretching, and carboxylic acids as the dominant groups in EPS. The in vitro DPPH assay of EPSs from PBBSCs showed 74.3% scavenging activity. Furthermore, the reducing power was determined to be 0.59 ata 500 mg/mL concentration, respectively. The extracted EPSs from the biological soil crust flocculated Kaolin clay suspension maximum at 500 mg/mL. Consequently, the cyanobacterial strain and exopolysaccharide characterization from the sacred forest’s biological soil crust were analyzed for their bioactive potential, bio-crust diversity, and distribution.

## 1. Introduction

Numerous endemic flora, fauna, and valuable ecological organisms inhabit sacred groves, representing micro-level biodiversity hotspots. Sacred groves are fragments protected by religious communities; they have religious significance for the protecting community. The value of sacred groves is immense. They serve as repositories of the valuable gene pool in wild relatives of crops, rich medicinal plants, and numerous other essential species [1]. Microorganisms in communally protected sacred forests could be due to soil moisture, temperature, minerals, and the availability of organic matter [2]. In areas exposed to sunlight, biological soil crusts form and play a crucial role in carbon fixation, nitrogen fixation, soil stabilization, and water retention. The cyanobacterial crust promotes the growth of floral species in the sacred forest by incorporating nitrogen, carbon, and plant growth promoters into the soil [3]. In addition to these soil nutrients, numerous species of cyanobacteria in the biological soil crust can synthesize EPSs. According to [4,5], cyanobacteria produce both external and internal polysaccharides. EPSs in the biological soil crust can absorb and retain water, forming a slimy film around cells and regulating water uptake. EPSs play a protective role in the survival of cyanobacteria in stressed habitats exposed to UV radiation, desiccation, and high temperature. The biological soil crust is abundant in cyanobacteria, which enhances the growth of other beneficial soil microorganisms, improves the carbon content of the soil by 75%, and increases the water-holding capacity. Industrial applications for cyanobacterial EPSs include bioflocculants, biopolymers, bioadhesives, and soil conditioners. The EPSs of cyanobacteria contain multiple functional groups (carbonyl, carboxyl, hydroxyl, and sulfate), which can bind to metal ions for their growth as well as nitrogen fixation. Cyanobacteria that fix nitrogen and solubilize phosphorus play a crucial function in nitrogen mobilization and phosphorus solubilization for plant growth [6].

Simple mechanical processes, such as a vortex, centrifugation, French press, and density gradient centrifugation, can typically be utilized to eliminate EPSs from the biological soil crust. EPSs from cyanobacteria confer health benefits such as antioxidant activities, anti-biofilm, immune modulator, and antitumor agents [7,8,9]. It is necessary to develop safe antioxidants from natural resources to minimize oxidative damage in living cells [10]. Exposure to ultraviolet (UV) radiation can generate reactive oxygen species (ROS) that damage biomolecules in cells and tissues. Natural antioxidants such as sterols, polyphenols, and flavonoids are widely used to reduce these effects [11]. Polysaccharides from natural products have multiple therapeutic properties. The challenge for researchers is to identify natural antioxidants that are safer and less toxic than synthetic antioxidants. In recent years, cyanobacteria polysaccharides have been found to contain significant amounts of neutral sugar and uronic acid, which can be utilized as bioflocculants [12].

A wide range of cyanobacteria has attracted the attention of researchers due to their capability to secrete a large quantity of EPS. The biological soil crust is the potential source for screening polysaccharide-producing cyanobacteria, which can be commercially exploited in industries for making gums, food additives, soil stabilizers, and the bioremediation of contaminated water [13]. This study aimed to (i) document prevailing cyanobacterial species in the biological soil crust from the sacred forest of Ariyalur and Pudukottai district of Tamil Nadu and (ii) examine EPSs in biological soil crusts and analyze their biochemical propertiesand antioxidant and flocculation activities.

## 2. Materials and Methods

### 2.1. Sample Collection and Identification of Cyanobacteria in the Biological Soil Crust

**Figure 1:**
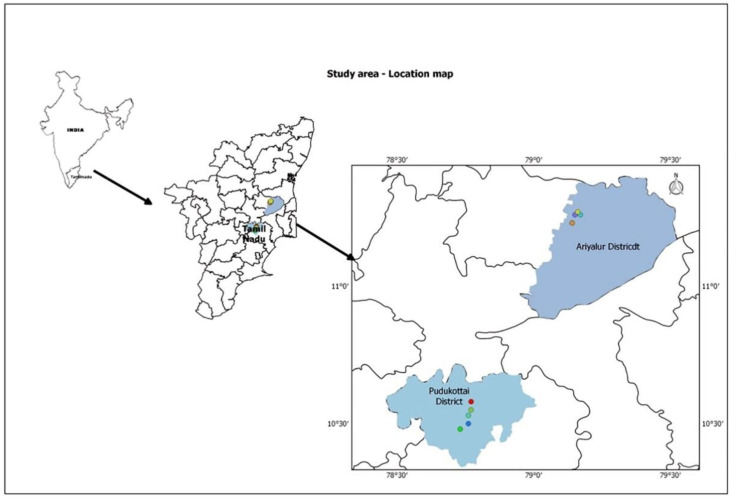
Geographical locations of sampling sites in Ariyalur and Pudukottai districts of Tamil Nadu, India. Each color represents the sacred grove areas within the study site.

The cyanobacterial biological soil crusts were collected from the sacred forest groves of Ariyalur and Pudukkottai districts of Tamil Nadu, India. Figure 1 depicts the study area, encompassing ten sacred forest groves located at 11°11′ N, 79°10′ E and 10°29′ N, 78°44′ E in the districts of Ariyalur and Pudukottai.

**Figure 2:**
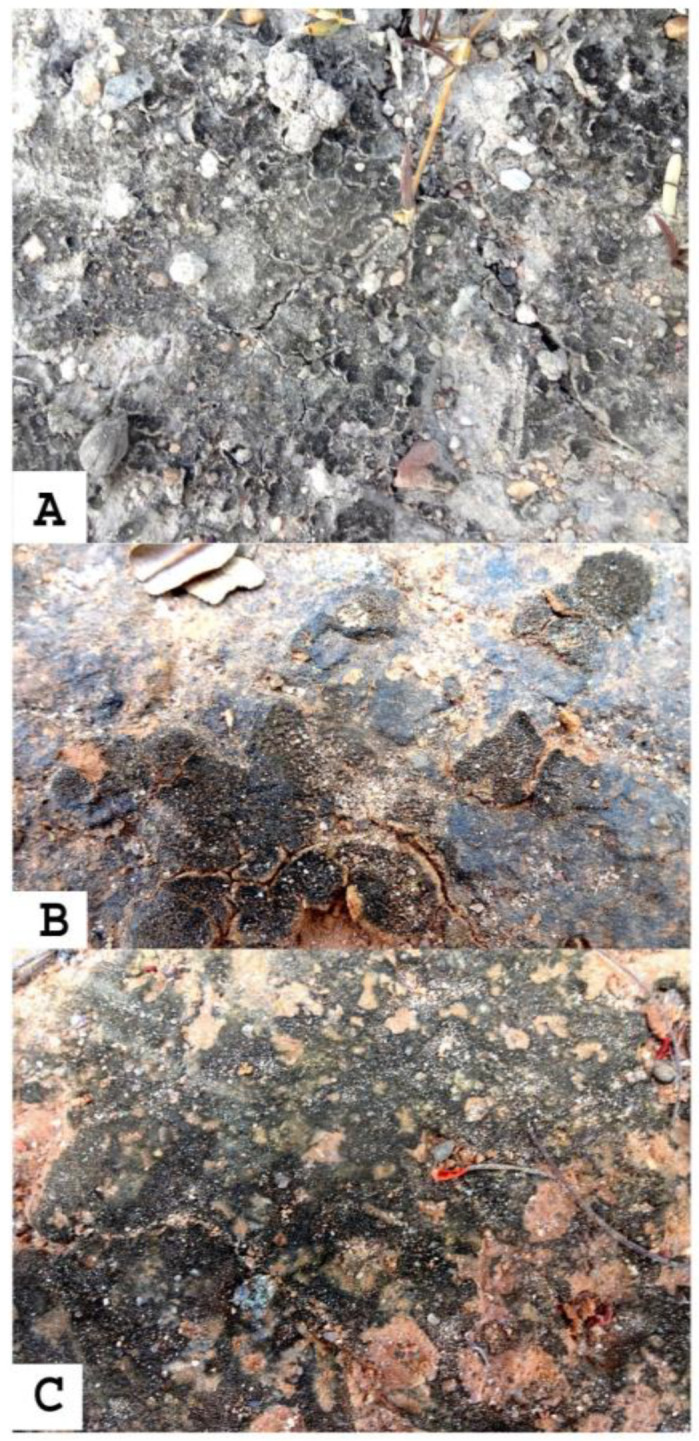
The appearance of blackish biological soil crusts in the sacred forests of Ariyalur and Pudukottai. ((**A**) Crust like blackish biological soil crusts, (**B**) Patches like blackish biological soil crusts, (**C**) Mat like blackish biological soil crusts) (**A**) blackcrusts, (**B**) greencrusts, (**C**) pinkcrusts.

We observed lichen, mosses, and a biological soil crust associated with cyanobacteria at the study site. Cyanobacterial crusts were prevalent and frequently observed on bare soil directly exposed to sunlight. Due to the presence of pigment-producing cyanobacteria, these biological soil crusts have various hues, including black, green, and pink. The black soil crust which predominates in the sacred forest groves of Ariyalur and Pudukkottaihas been examined. The blackish soil crusts collected from sacred forest groves of Ariyalur—ABBSCs (Ariyalurblackish biological soil crust) and Pudukkottai—PBBSCs (Pudukottaiblackish biological soil crust) are closely associated with soil surface (Figure 2).

The crust samples were collected during the early summer season. The study sites were rich in dry and deciduous vegetation. The biocrust samples were collected from 10 different sites in the study area. All the crust samples were subsequently stored in a desiccator at 37 °C. Biological soil crusts of different types, thicknesses, colors, and appearances in the field were recorded. A pinch of collected crusts was rehydrated for 2 h and observed under a compound microscope. The cyanobacteria were identified using the Desikachary method [14]. The cyanobacteria in the biological soil crust were isolated using serial dilution and spread plate techniques.

### 2.2. Genome Sequencing and Phylogenetic Analysis

The dominant cyanobacterial species were isolated and 16S rRNA gene sequences were amplified by PCR using the 518F universal forward primer 518F CCAGCAGCCGCGGTAATACG and the 800R reverse primer 800R TACCAGGGTATCTAATCC. These primer sequence data were aligned and analyzed for sample identification. The sequence similarity of 16S rRNA with the existing database was analyzed using the searching toolBLAST [15]. Using the BLAST search tool, the sequence similarity between 16S rRNA and the existing database was analyzed. The evolutionary relationships among microorganismswere studied using the phylogenetic trees constructed utilizing sequence data from the neighbor-joining and UPGMA methods. The distance of the branch was computed following the P-distance method, and the PhyML 3.0 Alrtprogram was used for phylogeny analysis [16].

### 2.3. Exopolysaccharide Extraction

EPS was extracted from the crust utilizing the modified method of Parikh and Madamwar [4]. A 10 g sample of biological soil crust was homogenized in a pestle and mortar to release EPS, and then suspended in 20 mL of water. Suspension centrifugation was performed for 30 min at 10,000 rpm under 4 °C. The supernatant was then kept overnight with 5 volumes of 80% acetone for precipitation at 4 °C. After 10 min of centrifugation at 12,000 rpm, the gel-like precipitate was collected and dissolved in deionized water. The suspension was dialyzed (dialysis membrane 110 kDa, Himedia, Maharashtra, India) overnight at 4 °C with 5 volumes of distilled water. After dialysis, the precipitate was successively washed with diethyl ether and then freeze-dried for further analysis.

### 2.4. Protein and Carbohydrate Analyses of EPS

The EPS protein concentration was determined following Lowry’s method, utilizing bovine serum albumin (BSA) as a standard measure at 595 nm [17]. The modified Anthrone method was employed to determine the carbohydrate content using glucose as a standard, measured at the wavelength 490 nm [18].

### 2.5. Physical Characterization of EPS

#### 2.5.1. Fourier Transform Infrared Spectroscopy (FTIR)

Functional groups in the polysaccharide were partially identified using an FTIR Spectrophotometer (Perkin Elmer Shelton, Waltham, MA, USA) [19]. Sample preparation was performed using a KBr pellet with EPS at a ratio of 2:1 *w*/*w*. The FTIR spectrum was verified at a range of 400–4000 cm^−1^.

#### 2.5.2. Liquid Chromatography–Mass Spectrometry (LC-MS)

The LC-MS Shimadzu LC-8040 system was utilized for determining the identification and separation of sugar compounds in the EPS. Lyophilized EPS of the 20 mg sample was hydrolyzed by mixing with 2 mL of 0.5 M trifluoroacetic acid and kept at 100 °C in a water bath for 4 h. The mixture was treated with diethyl ether before lyophilizing. The suspension was concentrated using a rotary flash evaporator at 60 °C to remove solvents. This concentrated EPS solution was dissolved in distilled water and analyzed for monosaccharide using LC-MS. The culture filtrate of EPS isolates was determined in a Shimadzu LC-8040 system with an injection volume of 10 μL. Subsequently, mobile-phase acetonitrile and water (80:20) were used for the separation in a C18 reverse-phase column. Positive and negative ion modes of an LC-8040 mass spectrometer with an electrospray ionization interface (ESI) were used to determine the masses of the separated fractions.

#### 2.5.3. Antioxidant Assay

##### 2,2-Diphenyl-1-picrylhydrazyl Assay (DPPH)

The scavenging activity of EPS from the biological soil crust at different concentrations was determined based on Hou et al.’s method [20]. Here, 1 mL of EPS extract was added to 1 mL of 0.002% 2,2-diphenyl-1-picrylhydrazyl (DPPH). The suspension was thoroughly mixed and kept in a dark room at 37 °C for 30 min. At 517 nm, the absorbance of the solution was measured with a spectrophotometer. Ascorbic acid served as the standard, and the following equation was used to calculate the scavenging potential:

$$DPPH\;radical\;scavenging\;ability=\left(\frac{1-OD\;of\;sample}{\;OD\;of\;control}\right)\times 100$$

##### Ferric Ion Reducing Antioxidant Power Assay (FRAP)

The reducing ability of EPS extracted from the biological soil crust was determined using the method of Benzie and Strain [21]. EPSs at various concentrations (100–500 μL) and standard ascorbic acid were added to 2.5 mL of 0.2 M sodium-phosphate-buffered solution (pH 6.6). Approximately 2.5 mL of potassium ferricyanide solution 1% (*w*/*v*) was added and kept at 50 °C for 30 min in a water bath. The suspension was added to 2.5 mL of 10% (*w*/*v*) trichloroacetic acid (TCA) before centrifugation at 3000 rpm for 10 min. Then, the supernatant (100 μL) was added to distilled water (100 μL) as well as 500 μL of FeCl_3_ 0.1% (*w*/*v*). Solution absorbance was read at 700 nm. Higher absorbance indicated the greater reducing potential of the samples.

#### 2.5.4. Flocculation Activity

EPS flocculation properties were analyzed by sedimenting the fine powdered Kaolin at a concentration of 4 g/L in distilled water. This suspension was spiked with different concentrations of EPS, vortexed for 5 min, and kept on standby at an ambient temperature (37 °C) for 10 min. At 550 nm, the absorbance of the 1.5 mL upper clear solution was determined using a spectrophotometer. The following equation was utilized for calculating the flocculation activity using distilled water as the control.

$$Flocculating\;property\;\left(\%\right)=\left(\frac{B-A}{B}\right)\times 100$$

where A is the absorbance of the sample at 550 nm, and B is the absorbance of the control at 550 nm.

### 2.6. Statistical Analysis

SPSS software and Origin 6.0 version were used for data analysis. Data are expressed as the mean ± standard deviation of three replicates.

## 3. Results

**Figure 3:**
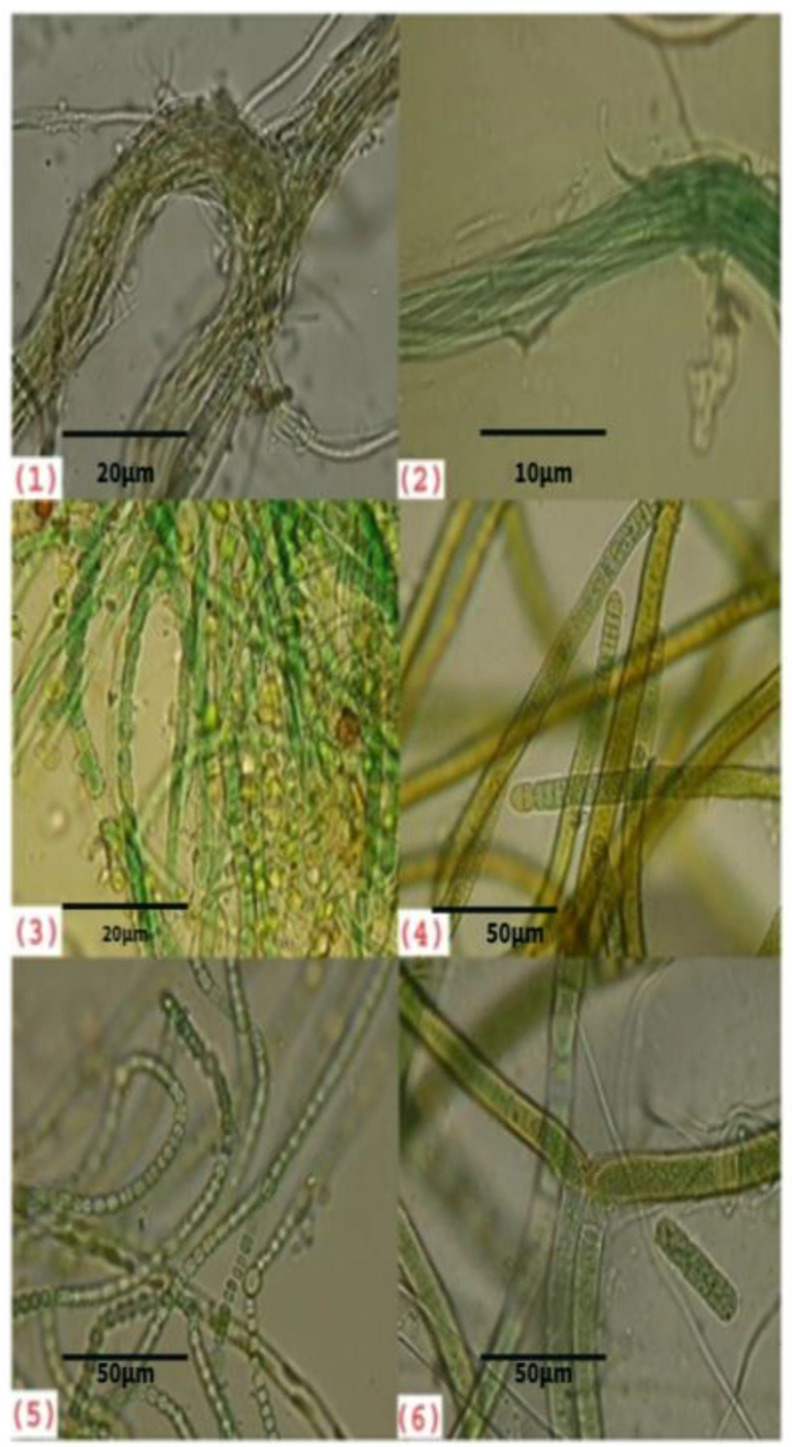
Photomicrographs of cyanobacteria from blackish biological soil crust in sacred groves. (**1**) *Microcoleusacutissimus*, (**2**) *Microcoleusorissia*, (**3**) *Anabaena* sp., (**4**) *Oscillatoria sancta*, (**5**) *Nostocpunctiforme*, and (**6**) *Scytonemaarcangeli*.

The cyanobacteria *Oscillatoria sancta*, *Nostocpunctiforme*, and *Scytonemaarcangeli* are dominant in the biological soil crusts of sacred forest groves (Figure 3). In all the biological soil crust, filamentous heterocyst and non-heterocystouscyanobacteria were in abundance. The present investigation reported that ABBSCs and PBBSCs of sacred forest groves exhibited 25 species of cyanobacteria listed in Table 1. In ABBSCs, there were 15 species of cyanobacteria, and in PBBSCs, 10 species of cyanobacteria were identified.

**Figure 4:**
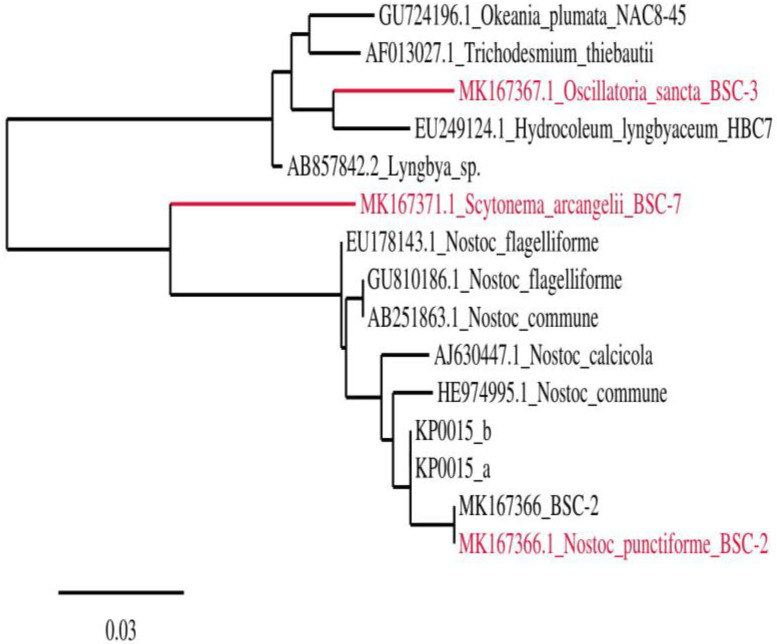
Evolutionary relationship of cyanobacteria in the blackish biological soil crust and red color indicated that the closely related dominant isolates are *Scytonemaarcangeli* MK167371.1, *Oscillatoria sancta* MK167367.1, and *Hydrocoleumlyngbyaceum* EU249124.1.

The 16S rRNA gene sequences of *Scytonema* sp. MK167371.1 and *Nostoc* sp. had the closest similarity to *Oscillatoria sancta* MK167367.1, closely related to *Hydrocoleumlyngbyaceum* EU249124.1, respectively. The distances in the tree generated by 16S rRNA gene sequencing specified the evolutionary relationships between the documented dominant strains *Scytonemaarcangeli*, *Nostocpunctiforme*, *Phormidiumautumnale*, *Oscillatoria sancta*, *Anabaena cylindrical*, *Calothrixdesertica*, and *Microcoleusacutissimus* originating from different geographical sites. The dendrogram clustering partially reflected the evolutionary relationships between the organisms (Figure 4).

### 3.1. Protein and Carbohydrate Analyses of EPSs

**Figure 5:**
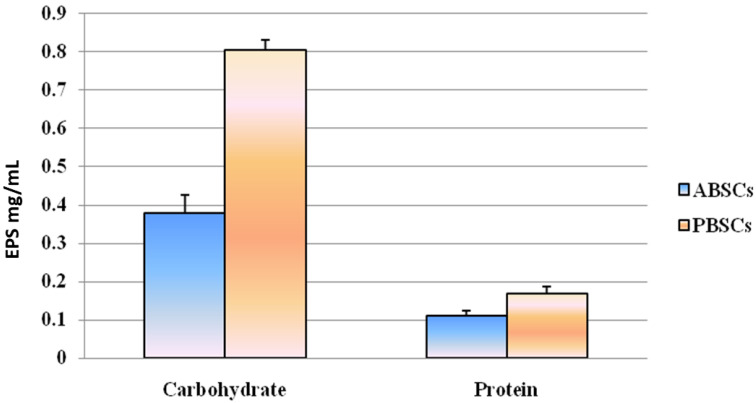
The protein and carbohydrate analyses of EPS from sacred grove biological soil crusts.

Cyanobacteria produce EPSs as a protective mechanism when exposed to extreme environmental conditions. The cyanobacterial crust (10 g) from the sacred forest grove yielded 0.14 g of exopolysaccharide. The crude EPSs isolated from the biological soil crust ABSCs resulted in 0.1093 ± 0.015 mg/mL, whereas EPSs of PBSC’s biological soil crust resulted in 0.1687 ± 0.019 mg/mL dry weight of protein content. In addition to the carbohydrate level in the EPS obtained from the biological soil crust, 0.3783 ± 0.048 mg/mL ABSCs and 0.8056 ± 0.025 mg/mL PBSCswere acquired. The biochemical analysis of EPS showed the presence of a significant amount of total carbohydrates and soluble proteins (Figure 5). The protein and carbohydrate levels of EPS from Ariyalur and Pudukottaibiological crusts were compared using ANOVA. The *p*-value was 0.307178–0.423969, which shows that the study variables were not statistically significant at *p* = 0.05. Thus, the resulting EPSs obtained from the two different biological crusts did not greatly influence the concentrations of protein and carbohydrate.

### 3.2. FTIR

**Figure 6:**
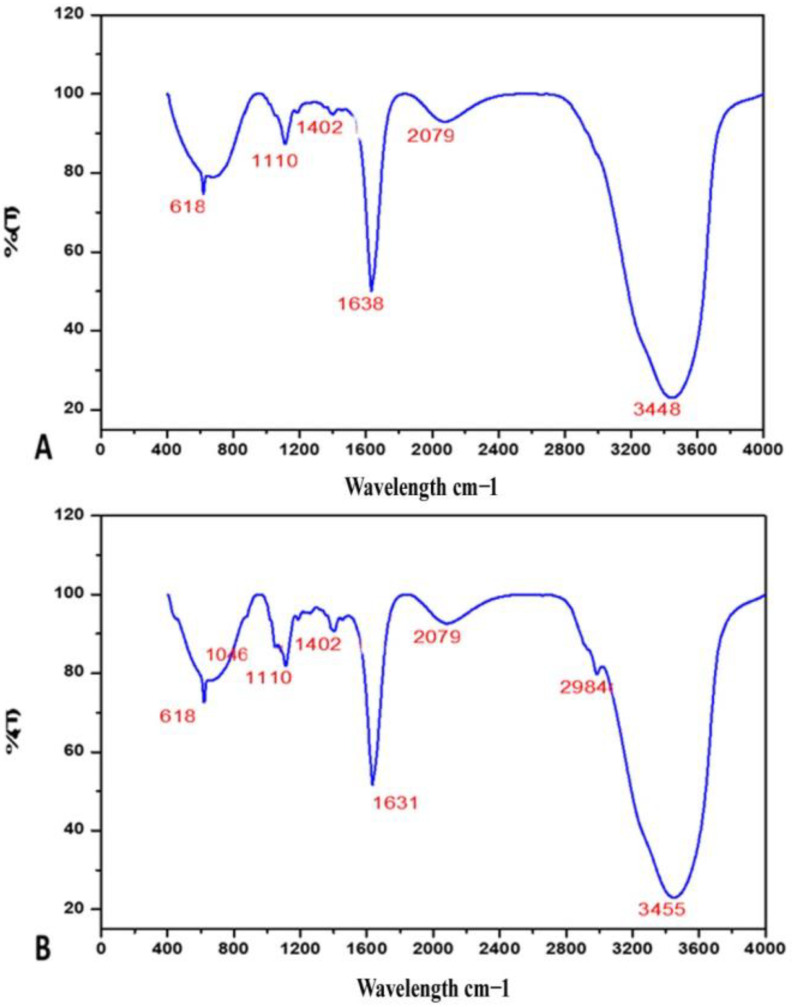
FTIR analysis for EPS extracted from sacred groves: (**A**) ABBSCs; (**B**) PBBSCs.

The FTIR spectra of extracted EPS from the biological soil crust revealedbroad, significant absorption peaks at 3455 cm^−1^ and 3448 cm^−1^,attributed to stretching vibrations of the amine (–NH) or hydroxyl (–OH) groups (Figure 6); peaks at 1110 cm^−1^ indicated the –CO stretch. The biological soil crusts of Ariyalur and Pudukottaiexhibited absorption peaks at 2984 cm^−1^, corresponding to the characteristic C-H stretch band, indicating that all peaks were characteristic of carbohydrates. The wide peak at 2079 cm^−1^ corresponded to the N=C=S stretch vibration for isothiocyanate. The peaks at 1402 cm^−1^ were due to the presence of the –OH groups of alcohol, carboxylic, or phenolic groups, whereas the peak at 1046 cm^−1^ corresponded to the –CN stretch, revealing the presence of an aliphatic amine group. The peaks at 1631 cm^−1^ and 1638 cm^−1^ corresponded to C=C stretching in the alkene groups.

### 3.3. LC-MS

**Figure 7:**
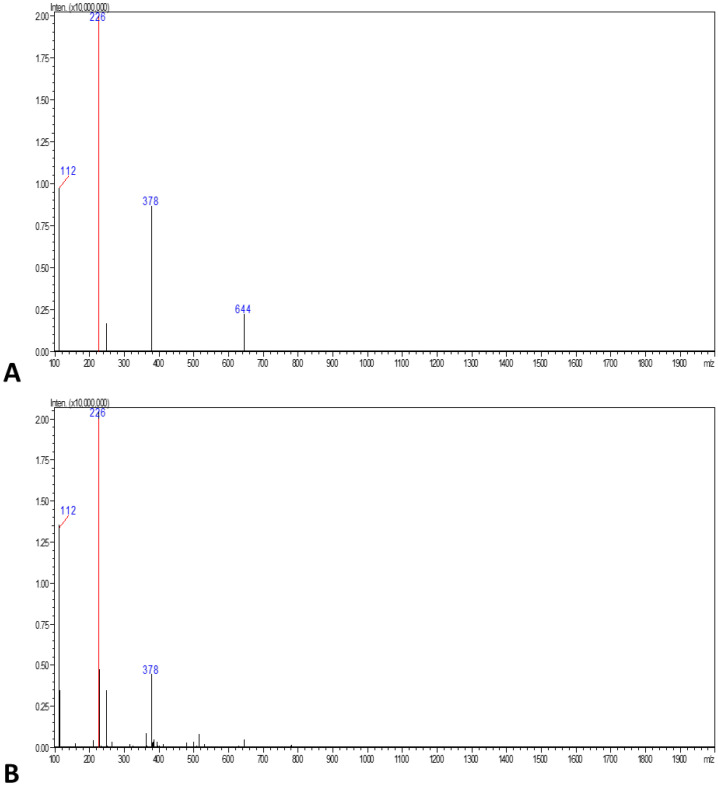
LC-MS analysis of EPS extracted from sacred groves: (**A**) ABBSCs; (**B**) PBBSCs. The mass value 226 for monasaccharides (glucose, fructose, galactose).

The hydrolyzed EPS monosaccharide composition was analyzed by LC-MS and the sugar residues (Figure 7). The peak value of the samples was charted and compared with the standard RF value. LC-MS analysis of hydrolyzed EPS samples indicated the presence of ribose, mannose, or glucose, and uronic acid as neutral sugars. The sugar components identified by LC-MS indicated that the EPSs extracted from the biological soil crust sample were likely glucose residues. The analysis of the sugar composition of EPS from ABBSCs and PBBSCs revealed that glucose was the predominant monosaccharide.

### 3.4. Antioxidant Activity

**Figure 8:**
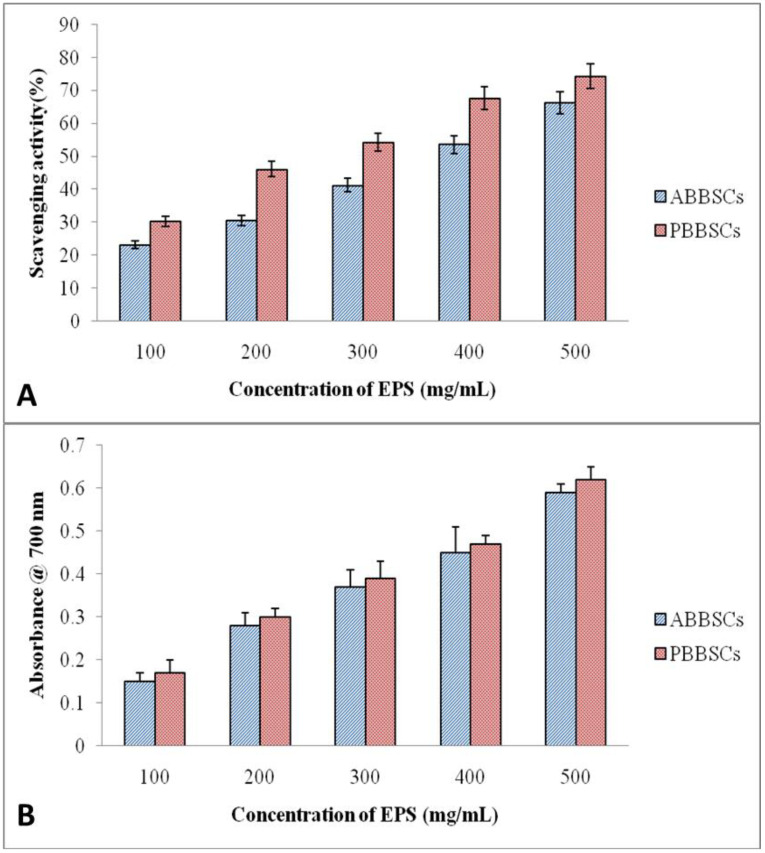
The antioxidant property of EPS from sacred grove biological soil crusts: (**A**) DPPH assay; (**B**) Reducing power assay.

By scavenging the free radical DPPH, the EPS antioxidant capacity was determined. Different concentrations (100 µL–500 µL) of EPS and ascorbic acid were examined for their ability to scavenge free radicals (DPPH). Figure 8B depicts the DPPH radical scavenging activity of EPSs from biological soil crust in comparison to ascorbic acid. We observed the concentration-dependent scavenging activity of isolated EPS. At 500 g/mL, PBBSC crust EPSs exhibited the highest scavenging activity, 74.3%, whereas ABBSC crust EPSs exhibited 66.2%. Increasing EPS concentrations tend to boost the reducing oxidation capacity. This result demonstrates that EPS is a good electron donor for stopping the chain reaction of free radicals. At a 500 µg/mL concentration, the absorbance of ABBSCs was 0.59 ± 0.02, and the absorbance PBBSCs was 0.53 ± 0.03, indicating the EPS reducing power. These results were compared with the absorbance of the standard antioxidant ascorbic acid, which showed an absorbance of 1.8 ± 0.01 (Figure 8A).

### 3.5. Flocculation Property

**Figure 9:**
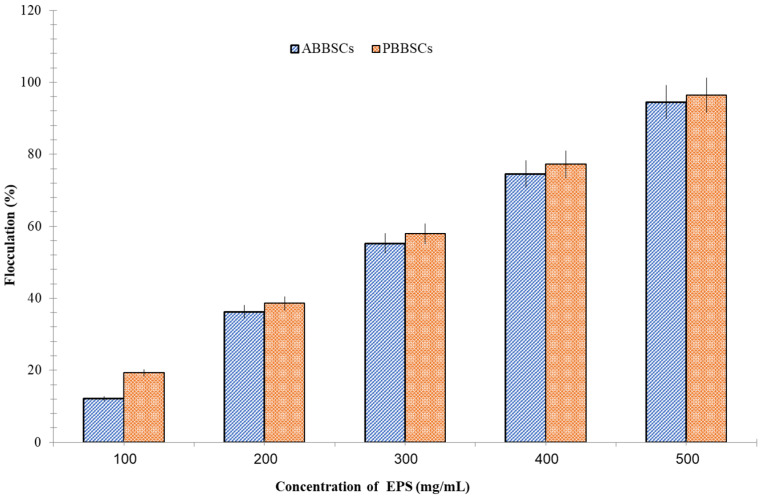
Flocculation activity of EPSs from sacred grove biological crusts.

It was observed that EPSs from the biological soil crust have flocculating activity (Figure 9). EPS induced the flocculation of fine kaolin particles and enhanced the flocculating process by increasing the concentration of EPSs. EPSs of biological soil crusts ABBSCs and PBBSCs at 500 mg/mL concentration showed ideal bioflocculant properties. The EPSs of PBBSCs exhibited greater flocculating activity than the EPSs of ABBSCs.

## 4. Discussion

Since the Convention on Biological Diversity (CBD) declaration, the significance of sacred groves in nature conservation has increased substantially [22]. This study demonstrated the cyanobacterial communities of the biological soil crust and EPSs at two sites in Tamil Nadu’s sacred forest groves, Ariyalur and Pudukottai. Cyanobacteria are photosynthetic light-sensitive and pigment producers [23], which give different colors to the crust. The microbial population in the biological soil crust significantly contributes to the regulation of nutrient recycling and energy flow in the ecosystem [24]. The species composition in the crust varied based on the physico-chemical composition of the soil, temperature, and light intensity. When analyzing the biological soil crust cyanobacterial communities from the sacred forests of different areas, we found that they were primarily composed of *Scytonemaarcangeli*, *Nostocpunctiforme*, *Phormidiumautumnale*, *Oscillatoria sancta*, *Anabaena cylindrica*, *Calothrixdesertica*, and *Microcoleuslacutissimus*. These results contradict a previous study on the sacred groves of Pudukottai and Ariyalur [25].

Phylogenetic analysis of cyanobacteria from blackish biological soil crust was performed. These cyanobacteria in the biological soil crust are primary producers of exopolysaccharides, which protect the organism from the harsh environment. EPSs from the biological soil crust increase the soil’s stability and are the primary contributor to further EPS synthesis. EPSs are biopolymers with significant ecological, industrial, and pharmaceutical importance [26]. EPSs released from cyanobacteria are composed of homopolysaccharides, which have repeated monosaccharides. In some cases, they contain heteropolysaccharideswith two or more distinct sugars. These sugars are arranged in linear or branched forms, substituted with methyl or sulfate groups. Microalgal EPSs have many biological activities, such as antibacterial, antiviral, antitumor, and antioxidant [27].

Extracellular polysaccharides enhance the soil’s texture, nutrient retention, and water-holding capacity [28]. Our findings demonstrate that EPS-producing cyanobacterial crusts are the most influential factor in restoring the sacred forest ecosystem’s fertility. The nutrient composition of the soil influences the diversity and dominance of cyanobacteria within the biological soil crust [29,30].

The dominant cyanobacteria identified in the bio-crust agree with prior reports of arid bio-crusts [25]. The cyanobacteria *Oscillatoria* sp., *Lyngbya* sp., and *Microcystis* sp. could produce EPSs [31], aligned with our present findings. In the present study, EPSs from different biological soil crusts showed the presence of carbohydrates and proteins. Hence, carbohydrates and protein were analyzed to determine the contribution of carbon and nitrogen to the soil. This report shows that carbohydrates usually dominate in EPS composition due to aging stress. Some microalgae produced a significant amount of EPS at the end of the growth phase. The accumulation of this polymeric substance causes flocculation [32]. EPSs produced by cyanobacteria envelope the cell in the form of a sheath, glycocalyx, capsule, or slime, rendering protection from physical and biological stresses [33,34]. This coincides with the findings of Chamizo et al. [35], who stated that cyanobacteria produce EPS and improve soil fertility by increasing C and N contents. The EPS produced by various cyanobacteria differed in their chemical composition [36]. The major composition of EPS is a carbohydrate with a small portion of proteins. Similarly, Redmile-Gordon et al. [37] stated that protein and carbon content was higher in the EPS of biological soil crust.

The functional groups in EPS correspond to Vicente-García et al.’s earlier reports [38]. The presence of aromatic, alkene, alkyne, alcohol, and carbonyl groups indicates the functional groups of cyanobacterial EPS reported by Khattar et al. [39]. The presence of bands less than 1000 cm^−1^ is probably due to the presence of potential linkages between monosaccharides. This characteristic absorption band of the carbohydrate ring is responsible for the solubility of EPS in water [40]. LC-MS analysis was utilized to confirm the functional groups of EPS. According to this study, EPSs from the biological soil crust were composed of monosaccharide glucose residues and uronic acids. Akaki et al. [40], Kim [41], and Ismail and Nampoothiri [42] reported that EPSs rich in monosaccharides showed good antioxidant and immunomodulation activities.

The sugar compounds in EPSs contribute to understanding biofilm formation and stabilization. Numerous studies have demonstrated that EPSs from various sources possess free radical scavenging properties. In EPSs, the functional groups, hydroxyl, phenol, and aliphatic amines, provide polymers with antioxidant properties and increased stability. The antioxidant property of EPS can donate electrons to stabilize radicals or react with free radicals to stop the reaction of the radical chain [43]. Based on the ferric-reducing power assay, the FRAP assay revealed the antioxidant potential of EPS. EPSshave a high reduction potential, indicating their potential antioxidant activity. Furthermore, the EPSs extracted from the biological soil crust produced FRAP values of a high order. In this study, the reducing power assay demonstrated the ability of EPS to absorb ferricyanide and convert it to ferric, which is colored Prussian blue [44]. Similarly, the methanol extract of EPS from cyanobacteria, Arthrospiraplatensis, showed antioxidant activity [45]. The soil’s antioxidant capacity monitors the organic matter breakdown rate which aid in the retention of soil organic matter.

The adhesion of microorganisms in biofilms and their establishment on the soil surface are the most critical functions of bioflocculants. Multiple types of macromolecular interactions, including hydrogen bonds, electrostatic interactions, and dispersion forces, mechanicallystabilize EPS microbial aggregates. The resultant formation of a gel-like three-dimensional structure around the cells enables the microorganisms to remain close to one another and form stable consortia [46]. Flocculation is an essential property of EPSs because water primarily moves through large pores between aggregates.

Additionally, plant roots primarily grow between aggregates. The maximum flocculation activity was recorded at 500 mg/mL (96%);the minimum activity was recorded at 100 mg/mL.This indicated that the flocculation activity of EPS was caused by the surface-charge-induced bridging mechanism. Positively charged EPSs decreased the negative electrical charge of the Kaolin, resulting in flocculates. EPSs with a positive charge decreased the Kaolin’s negative electrical charge, leading to flocculates. It was observed that EPSd from the biological soil crust induced the flocculation of fine kaolin particles and enhanced the flocculating process by increasing the concentration of uronic acid in the EPS [47,48]. In addition, the flocculation activity of EPSs is due to their natural anionic charge. In this study, for 1 L of kaolin (4 g) solution, a volume of 100 mL of crude EPS solution containing 50 g of EPS was added for maximum flocculation. If in purified form, we could obtain maximum flocculation at minimal concentration of EPS. The flocculation properties of EPS were greatly influenced by the cyanobacterial species. The EPSs extracted from cyanobacteria *Anabaena* sp. and *Nostoc* sp. exhibited comparable flocculation activity. EPSsare abundant in uronic acid, and have enhanced flocculent properties [49,50]. Bioflocculantshave wide applications in industrial and environmental applications. These polymers can absorb heavy metals from the soil. These flocculants cause soil aggregation and the accumulation of nutrients [51].

## 5. Conclusions

Approximately 25 cyanobacteria species were identified from the Ariyalur and Pudukottai sacred groves of Tamil Nadu, India. Members of Oscillatoriaceae, Scytonemataceae, and Nostocaceaewere densely populated in the biological soil crust from the sacred groves of Ariyalur. These nitrogen-fixing cyanobacteria in the sacred groves are attributed to the enhanced soil nitrogen level, promoting vegetation. The physicochemical analyses of EPSs of the biological soil crust revealed significant amounts of total soluble proteins and carbohydrates. These isolated EPS had a complex composition of neutral sugars such as glucose/mannose, uronic acid, and ribose. The FTIR profile indicated that EPSshad the following functional groups: phenol, aromatic, alkene, and amine. Some of the functional groups, such as the free hydroxyl and phenol groups, provide antioxidant properties. The in vitro antioxidant assay, DPPH, and reducing power assay showed that EPSs possess free radical scavenging activity. The flocculation of Kaolin clay caused by adding EPS shows its anionic nature and aggregation property. The isolated cyanobacterial EPS showed good antioxidants, an indicator of carbon pool, and flocculation activity dictating soil aggregation. EPSs from biological soil crust conferred their significance in the fertility of the sacred forest soil. The EPS-producing cyanobacterial crust in sacred forest groves conferred nutrient cycling and improved soil stability. Industrially, these EPS can also be a potential natural antioxidant and bioflocculant source. The documented cyanobacterial population from the biological soil crust is a potential restriction for the commercial production of EPS and biofertilizers. The utilization of cyanobacteria as an inoculant to promote biological crust development has been proposed as a novel biotechnological method for restoring degraded arid areas, as well as combating desertification.

## Figures and Tables

**Table 1:** Predominant cyanobacteria in the biological soil crust of sacred groves.

S.NO	Cyanobacterial Species	Types of Biological Soil Crust	
		ABBSCs	PBBSCs
1.	*Anabaena* sp.	−	+
2.	*Gloeocapsarupestris* Kutz.	−	
3.	*Hapalosiphonbaroni* West, W., and G., S.	+	−
4.	*Lyngbyakasyapii* Ghose.	+	−
5.	*Lyngbyalachneri* (Zimm.) Geitler.	+	−
6.	*Lyngbyarivularianum* Gom.	+	−
7.	*Microcoleusacutissimus* Gardner.	+	+
8.	*Microcystisaeruginosa* Kutz.	+	+
9.	*Microcystis* sp.	−	−
10.	*Nostocpunctiforme*	−	+
11.	*Oscillatoria sancta* (Kutz.) Gom.	+	−
12.	*Phormidiumfoveolarum* Gom.	−	+
13.	*Phormidiumabronema* Skuja.	−	+
14.	*Phormidiumangustissimum* West, W., and G., S.	−	+
15.	*Phormidiumbohneri* Sohmidle.	+	−
16.	*Phormidiumfoveolarum* Gom.	+	−
17.	*Phormidium* sp.	−	+
18.	*Phormidiumtenue* (Menegh.) Gom.	−	+
19.	*Phormidiumusterii* Schmidle.	−	+
20.	*Scytonemaarcangeli*	+	−
21.	*Scytonemajulianum* (Kutz.) Menegh.	+	−
22.	*Scytonemamillei* Born.	+	−
23.	*Scytonemapseudopunctatum* Skuja.	+	−
24.	*Scytonemaschmidtii* Gom.	+	−
25.	*Scytonema* sp.	+	−
	Total	15	10

(+ Species present in the designated area, − Species absent in the designated area).

## Data Availability

Not applicable.
